# *Locustamigratoria* (L.) (Orthoptera) in a warming world: unravelling the ecological consequences of climate change using GIS

**DOI:** 10.3897/BDJ.12.e115845

**Published:** 2024-03-05

**Authors:** Eslam M. Hosni, Areej A. Al-Khalaf, Mohamed G. Nasser, Sara M. ElShahed, Sara A. Alashaal

**Affiliations:** 1 Research Lab of Biogeography and Wildlife Parasitology, Department of Entomology, Faculty of Science, Ain Shams University, Cairo, Egypt Research Lab of Biogeography and Wildlife Parasitology, Department of Entomology, Faculty of Science, Ain Shams University Cairo Egypt; 2 Biology Department, College of Science, Princess Nourah Bint Abdulrahman University, Riyadh, Saudi Arabia Biology Department, College of Science, Princess Nourah Bint Abdulrahman University Riyadh Saudi Arabia

**Keywords:** Acrididae, environmental niche modelling, Geographic Information System (GIS), lobal warming, migratory locust, MaxEnt modelling

## Abstract

The migratory locust, *Locustamigratoria* (L.), a significant grasshopper species known for its ability to form large swarms and cause extensive damage to crops and vegetation, is subject to the influence of climate change. This research paper employs geographic information system (GIS) and MaxEnt ecological modelling techniques to assess the impact of climate change on the distribution patterns of *L.migratoria*. Occurrence data and environmental variables are collected and analysed to create predictive models for the current and future distribution of the species. The study highlights the crucial role of climate factors, particularly temperature and precipitation, in determining the locust's distribution. The MaxEnt models exhibit high-performance indicators, accurately predicting the potential habitat suitability of *L.migratoria*. Additionally, specific bioclimatic variables, such as mean temperature and annual precipitation, are identified as significant factors influencing the species' presence. The generated future maps indicate how this species will invade new regions especially in Europe. Such results predict the risk of this destructive species for many agriculture communities as a direct result of a warming world. The research provides valuable insights into the complex relationship between locust distribution and environmental factors, enabling the development of effective strategies for locust management and early warning systems to mitigate the impact on agriculture and ecosystems.

## Introduction

*Locustamigratoria* (L.), commonly known as the migratory locust or the African locust, is a species of grasshopper that is widely distributed across various regions of the world (Lecoq 2022). It belongs to the family Acrididae, which includes numerous grasshopper species. *Locustamigratoria* is one of the most notorious and economically significant locust species due to its ability to form large swarms and cause extensive damage to crops and vegetation (Schoofs et al. 1993; Raffini et al. 2020).

*Locustamigratoria* has a broad distribution range and can be found in many parts of the world, primarily in temperate, tropical and subtropical regions. Historically, its native range spans Africa, including areas such as northern and western Africa, the Arabian Peninsula and parts of Asia. However, due to its migratory behaviour and adaptability, the species has expanded its distribution to other continents as well (Malakhov et al. 2018). *Locustamigratoria* has been reported in countries across Europe, including Spain, Italy, France and Greece (Sergeev 2021). It has also been found in parts of Asia, including China, India, Pakistan and Japan (Sergeev 2021). In Australia, the species is known as the Australian plague locust and is considered a major agricultural pest (Hunter et al. 1999). Additionally, it has been a cause of concern for agricultural regions in New Zealand due to its ability to swarm and devour crops (Rieger et al. 2007).

The distribution and population dynamics of *L.migratoria* are influenced by various factors such as climate, vegetation and habitat availability. Favourable conditions, such as periods of rainfall and vegetation growth, can contribute to population booms and the formation of swarms (Jyoti et al. 2020). The ecology of this species is rarely studied and the way within which its population will behave to climate change is infrequently known (Chapuis et al. 2009). New techniques, such as remote sensing and geographical information systems, will greatly help in better understanding of these issues (ElShahed et al. 2023).

The Geographic Information System (GIS) allows researchers to integrate and analyse various spatial data layers, such as climate variables, vegetation indices, land cover and topography, to create comprehensive maps and models of organism distribution (Miller 2010). By examining the relationships between locust occurrence or abundance and environmental factors, GIS helps identify key variables influencing their distribution patterns. MaxEnt is a machine-learning algorithm commonly used in ecological modelling to predict species distributions (Merow et al. 2013). It uses presence-only species occurrence data along with environmental variables to estimate the probability of species occurrence across a landscape (Phillips et al. 2023). MaxEnt models have been employed to understand the environmental niche of several insect species and predict their potential distribution under different climate scenarios (Royle et al. 2012; Di Febbraro et al. 2023;ElShahed et al. 2023).

When studying the impact of climate change on *L.migratoria* distribution, the historical climate data and future climate projections could be used to generate distribution modelling maps to predict the distribution pattern of this species (Wei et al. 2023). By comparing the current distribution of the species with future climate scenarios, scientists can assess how changes in temperature, precipitation patterns and other climatic variables may affect the potential range and abundance of *L.migratoria*. These modelling approaches help researchers understand the complex relationships between locust distribution and environmental factors, enabling them to make informed predictions about future changes in locust range and identify areas that may be at higher risk of locust outbreaks due to climate change (Kalra et al. 2016). This information can be crucial for developing effective strategies for locust management and implementing early warning systems to mitigate the impact on agriculture and ecosystems (Blank and Blaustein 2012).

The hypothesis of this research posits that the distribution patterns of *Locustamigratoria* will be significantly influenced by the ecological consequences of climate change. By employing Geographic Information Systems (GIS) and MaxEnt ecological modelling techniques, the study aims to investigate the impact of climate factors, particularly temperature and precipitation, on the distribution of *L.migratoria*. The overarching prediction is that changes in these climate factors will have profound effects on the species' distribution. As climate change continues to manifest, it is anticipated that *L.migratoria* will experience an expansion of its range. The warming temperatures and altered precipitation patterns associated with climate change may create more favourable conditions for this locust species, allowing it to colonise previously unsuitable regions. Consequently, research predicts an increased likelihood of *L.migratoria* invading new areas, particularly in Europe, as a direct consequence of a warming world. Moreover, the study expects to observe shifts in the suitable habitat for *L.migratoria* due to climate change. As temperature and precipitation patterns change, the locations that were once suitable for the species may become less favourable, while previously unsuitable areas may become more conducive to its survival. These shifts in suitable habitat could have significant implications for the distribution and abundance of *L.migratoria* populations.

## Materials and Methods

### Occurrence data

**Figure 1.**
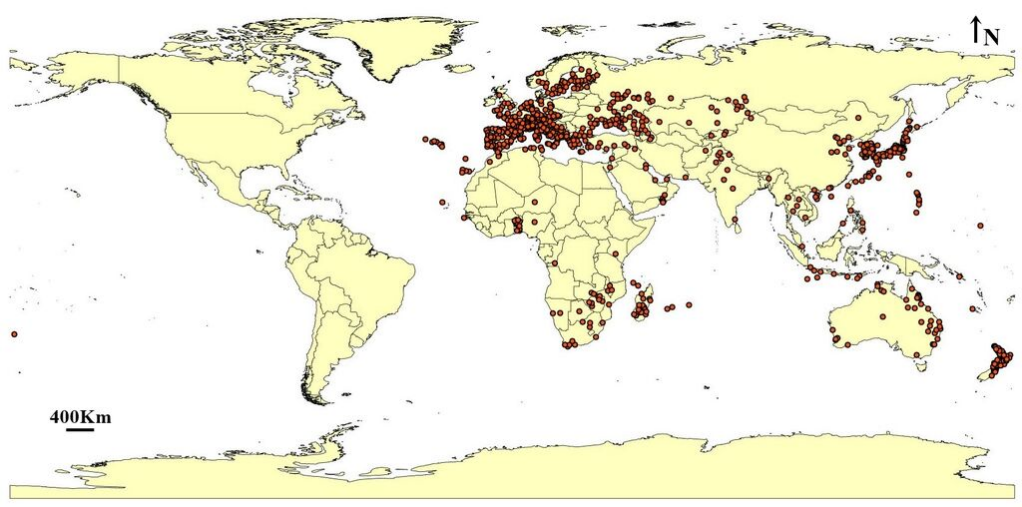
Natural distribution of *Locustamigratoria*.

Records were downloaded from the Global Biodiversity Information Facility database (www.gbif.org). Originally, 9,429 occurrence points were obtained for modelling. The data were filtered through three main steps. First, removal of points without longitudes and latitudes; second, deletion of duplicated records and third, spatial rarefaction, based on 50 km distance using ArcGIS v. 10.3 (SDM toolbox: SDM tools; Universal tools—Spatially rarefy occurrence data) (Hosni et al. 2022). A total of 820 unique points were converted to CSV format and used to model the current and future potential distribution of *L.migratoria*. The insect is mainly concentrated in western Europe, Japan, New Zealand and some parts of Africa and Australia (Fig. 1).

### Environmental data

Nineteen bioclimatic variables accounting for temperature and precipitation were attained from the WorldClim database (ww.worldclim.org). The variables have a spatial resolution of 2.5 arc-min (5 km^2^). These are monthly climatic data for the years 1950-2000 collected by forecast stations.

For the current prediction model, the bioclimatic variables 8-9 and 18-19 were excluded because of spatial inconsistencies that affect their resolution (Escobar et al. 2014, Hosni et al. 2020). The remaining fifteen variables were converted to ASCII format using ArcGIS v. 10.3. To remove the correlation between variables, Pearson’s correlation coefficient was used at a value equal to r^2^ ≥ |0.8|. In addition, multicollinearity was reduced by the SDM toolbox function in ArcGIS v. 10.3 (universal tool; explore climate data; remove highly correlated variables) (Hosni et al. 2022). The five most significant bioclimatic variables were taken for further analysis.

Regarding the future prediction model, a corresponding set of variables was also downloaded from the WorldClim database for the periods 2050 and 2070, for the representative concentration pathways (RCPs) 2.6 and 8.5. RCPs represent projections of radiative forcing components that are created as input to climatic models, with RCP 2.6 being the lowest emission scenario and RCP 8.5 being the highest (van Vuuren et al. 2011). After conversion to ASCII format in ArcGIS v. 10.3, the layers were used in the future model.

Three General Circulation Models (GCMs) were used for both RCPs (2.6 and 8.5) for the periods 2050 and 2070. The GCMs are the Beijing Climate Center (BCC-CSM 1_1), the National Center for Atmospheric Research (CCSM4) and the Meteorological Research Institute (MRI-CGCM3). Then, the mean of the three GCMs was calculated for each RCP for both 2050 and 2070 for comparison with the current distribution model. Furthermore, loss and gain in habitat suitability were estimated using the map algebra function of ArcGIS v. 10.3.

### Modelling approach

*Locustamigratoria*'s current and future distribution was modelled using the MaxEnt software tool v. 3.4.1 (Phillips et al. 2023). It forecasts the existence of a species by combining occurrence records with background data from environmental variables in the research region (Kumar et al. 2015; Zacarias 2020). MaxEnt ranks locations with conditions similar to their current occurrences from 0 (unsuitable) to 1 (suitable) (Kumar et al. 2015). It has been used successfully to forecast the prospective distribution of several insect species of medical and economic value in specific places as well as internationally (Aidoo et al. 2023, Al-Khalaf et al. 2023).

We divided the occurrence records into two sets, with 75% of the data used for training the MaxEnt models and the remaining 25% for testing. This split allowed us to assess the model's performance on independent data and evaluate its predictive ability. The choice of a 75%/25% split is a commonly employed practice in ecological modelling studies (Hosni et al. 2020). The number of iterations was set to 500. The number of iterations represents the number of times the MaxEnt algorithm adjusts model parameters to maximise the likelihood of occurrence data, given the environmental variables. Selecting an appropriate number of iterations is crucial for achieving model convergence and stability (Phillips et al. 2023). The number of background points was set to 10,000 which represent the environmental conditions in the study region. Background points are randomly generated pseudo-absences used to define the range of environmental conditions available to the species (Phillips et al. 2023). The selection of 10,000 background points ensured a representative coverage of the study area while maintaining computational feasibility (Hosni et al. 2022). To improve the model's performance, we used 10-fold cross-validation (Hosni et al. 2022). Cross-validation partitions the data into multiple subsets, training the model on a subset and evaluating it on the remaining data. By performing this process iteratively, we obtained an estimate of the model's performance across different subsets of data (Hosni et al. 2022). Using ArcGIS v. 10.3, regions were classified into five classifications based on habitat suitability (unsuitable, low, medium, high and extremely high) (Al-Khalaf et al. 2023). This classification allowed us to interpret and communicate the model outputs in a meaningful and easily understandable manner.

### Model evaluation

The Area Under the Curve (AUC) of the Receiver Operating Characteristics (ROCs) and the True Skill Statistics (TSS) were used to evaluate the resulting models (Salinas-Ramos et al. 2021; ElShahed et al. 2023). AUC values less than 0.5 were judged as poor fit, whilst values more than 0.75 were regarded as good fit (Phillips et al. 2023). TSS was calculated to assess model accuracy further (Hosni et al. 2022). Values near 0 indicated a weak association between the model and the distribution, whereas values near 1 indicated a high relationship (Hosni et al. 2022).

### Two-dimensional niche analysis and the most limiting factors

To analyse the two-dimensional niche of *Locustamigratoria*, we utilised Diva-GIS software. The Envelope test was applied, focusing on two key climatic variables: annual mean temperature (bio_1) and annual precipitation (bio_12) (Hosni et al. 2022). This test allowed us to assess the range and suitability of these variables for the species. Additionally, we employed the bioclimatic statistical modelling feature within Diva-GIS to generate a limitation factor map, based on the selected variables (Zhang et al. 2022). This approach enabled us to identify and map the factors that most significantly limit the distribution of *L.migratoria*, providing valuable insights into the ecological constraints affecting the species.

## Results

### Model assessment and contribution of environmental variables

**Figure 2.**
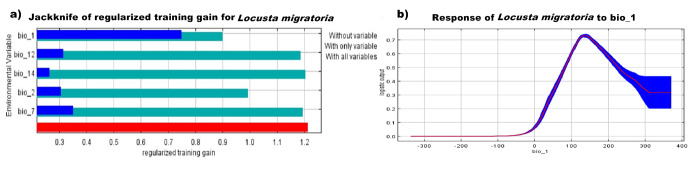
Maxent modelling outcomes. a) The Jackknife test of the most important variables for *L.migratoria*; b) Response curves of the most effective bioclimatic factor (bio 1) in the migratory locust’s distribution.

The TSS and the AUC were used as a measure for the accuracy and performance of our MaxEnt model. The model for *L.migratoria* showed a high AUC value of 0.89 which is an indicator of the model’s excellent performance (Suppl. material 1). We also calculated the TSS of the model which further confirmed its accuracy at a value of 0.75. The Jackknife test was conducted on the five most significant bioclimatic variables: bio 1 (Annual Mean Temperature), bio 2 (Mean Diurnal Range), bio 7 (Temperature Annual Range), bio 12 (Annual Precipitation) and bio 14 (Precipitation of the Driest Month) (Fig. 2). The test showed that bio1 (Annual Mean Temperature) is the most contributing variable at 56% and bio 2 is the second at 24.9%. Bio 12 and 14 followed with contribution percentages of 7.9 and 7.1, respectively. Moreover, the response curve indicated that the favourable temperature for *L.migratoria* ranges between 10°C and 15°C (Table 1, Fig. 2b).

**Figure 3.**
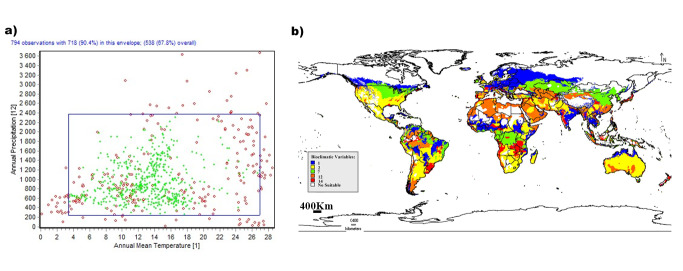
DIVA-GIS analysis of climatological variables and its effects on *L.migratoria*. a) Two-dimensional niche between Annual Temperature (bio 1) and Annual Precipitation (bio 12) for *L.migratoria*; b) Limitation factor map of the selected variables for *L.migratoria*.

The two-dimensional niche analysis indicates the high adaptability of this species towards the evaluated factors. It can live in a very harsh environment of the desert area and, on the other hand, in highly rainy and cold ones (Fig. 3), but through this huge range some climatological factors form a limitation for the life of migratory locusts by its fluctuation: the very low precipitation throughout the Middle East, very low temperature through most of Europe and very high diurnal temperature in Australian desert (Fig. 3).

### Predicted Current Potential Distribution of L.migratoria

**Figure 4.**
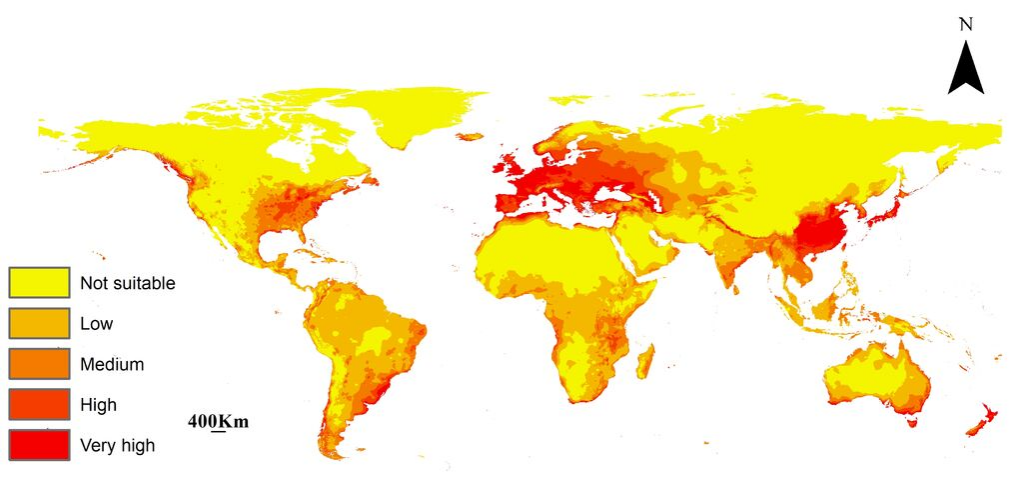
Predicted current global distribution for *L.migratoria*.

The current prediction model agrees with the natural distribution of the species in Europe, Africa, Asia and Australia. In Europe. It is mostly concentrated in the west and is found in the coastal areas surrounding the Baltic Sea, Black Sea, Mediterranean Sea, North Sea, Adriatic Sea and the Caspian Sea. The model is in accordance with the ecology and behaviour of *L.migratoria* as most outbreaks happen in the deltas of rivers flowing into these seas. This is clearly depicted in Africa in the northern regions of the Mediterranean coasts that have no occurrence records, but are predicted to be of very high suitability currently especially north of Egypt, Libya and Morocco. Additionally, low to medium habitat suitability is seen in southern Africa (Fig. 4).

In Asia, the model shows very high suitability in Japan and the southern regions of North Korea. Further, all of China is predicted to be at very high risk. Notably, the regions surrounding the Brahmaputra River are of very high suitability as well. Low to medium suitability is found in most of India, Singapore, and Indonesia. In Australia, the pest is found in the southeast and New Zealand and the model forecasts low to medium habitat suitability in some regions (Fig. 4).

### Predicted future distribution of L.migratoria

**Figure 5.**
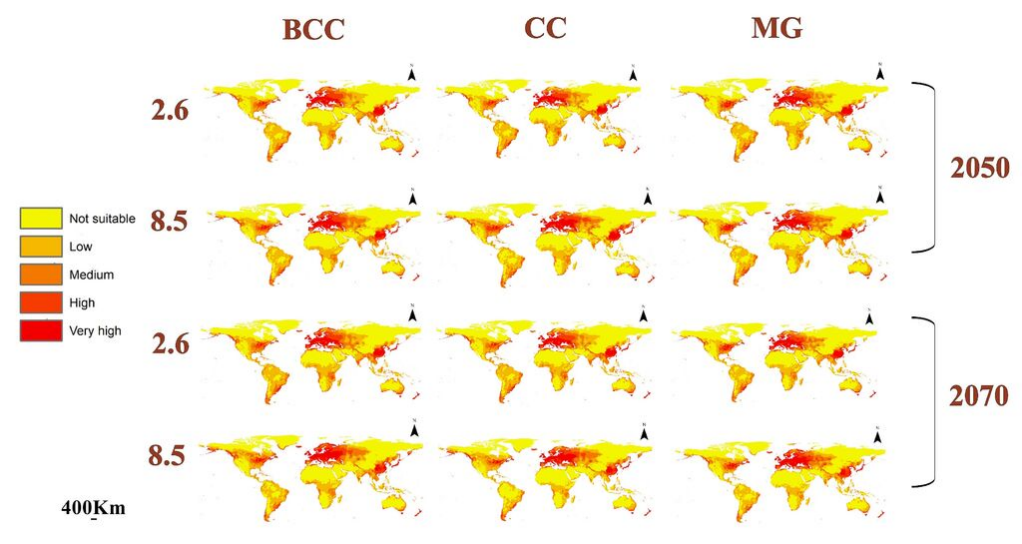
Predicted future distribution of *L.migratoria* in 2050 and 2070 under the RCPs 2.6 and 8.5 for the GCMs. BCC: Beijing Climate Center (BCC-CSM 1_1); CC: National Center for Atmospheric Research (CCSM4); MG: Meteorological Research Institute (MRI-CGCM3).

**Figure 6.**
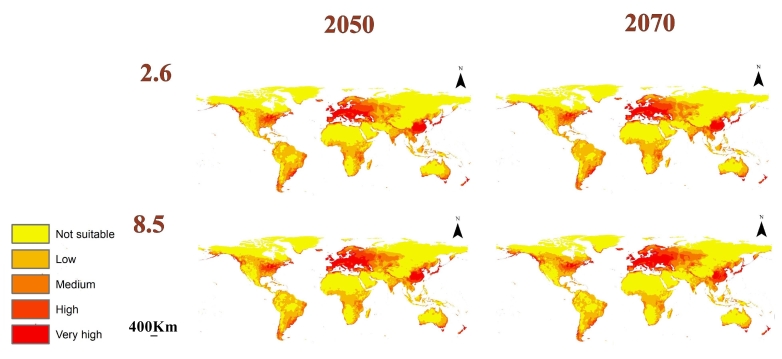
Calibration maps showing the mean of the three GCMs for the RCPs 2.6 and 8.5 in 2050 and 2070.

**Figure 7.**
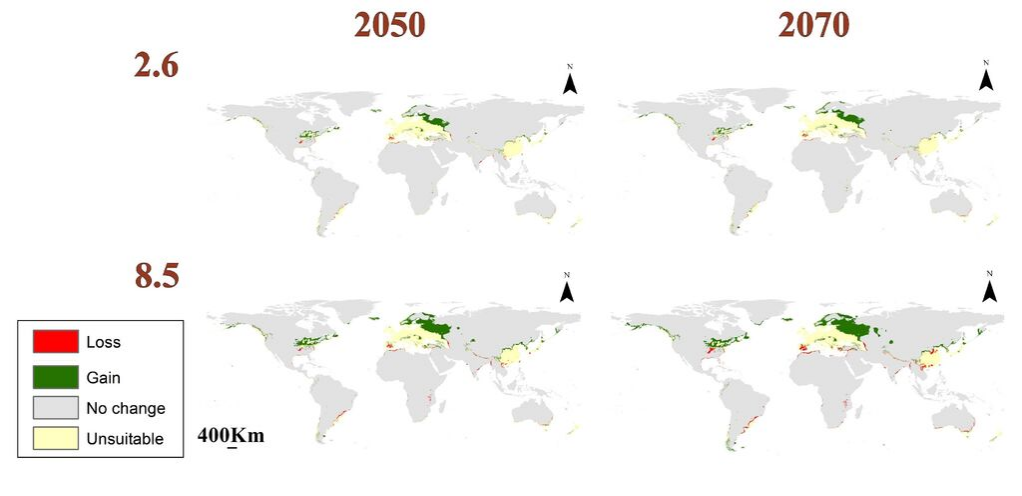
Map showing gain and loss in habitat suitability for the time periods 2050 and 2070 in comparison to the current prediction map.

We used three GCMs in the data of the future model considering the RCPs 2.6 and 8.5 in the time periods 2050 and 2070 (Fig. 5). The mean of the three GCMs was calculated to estimate the overall future changes in the habitat suitability of *L.migratoria* (Fig. 6). Additionally, the loss and gain in suitability were calculated to account for the differences between the current and future models (Fig. 7).

The majority of western Europe is predicted to be unsuitable for *L.migratoria*. However, a significant gain in suitability is visible in Norway, Sweden, European Russia and Finland. Moreover, we see a loss in suitability in China along with noticeable unsuitability in most of the country and some gain in certain regions. The majority of Japan is also forecast to be unsuitable, except for a region in the north where a gain is depicted. Furthermore, some regions in New Zealand become unsuitable while others show gain. In Africa, a loss in habitat suitability is found in Morocco. As for the Americas, a clear gain is found in the U.S. in the region of the Great Lakes which reaches a maximum at the RCP 8.5 2070 scenario. Further, a loss in eastern Brazil can be seen along with a gain in the south of Chile and the Falkland Islands (Fig. 7).

## Discussion

The present study focuses on modelling the current and future potential distribution of *L.migratoria*, commonly known as the migratory locust, using MaxEnt software and environmental variables. Understanding the ecological consequences of climate change on locust distribution is crucial, as it has broader implications for ecosystems and agriculture (Despland et al. 2003). The research seeks to elucidate the complex relationship between locust distribution and environmental factors, shedding light on how changes in climate can impact species interactions, biodiversity and ecosystem functioning. This knowledge is vital for the development of effective strategies for locust management and the establishment of early warning systems to mitigate the potential detrimental effects on agriculture and ecosystems (Al-Ajlan 2007). The results provide valuable insights into the factors influencing the distribution of this economically significant locust species and its potential response to climate change.

The evaluation of the MaxEnt model demonstrated its high accuracy and performance in predicting the distribution of *L.migratoria*. The high AUC value of 0.89 indicates excellent model performance, suggesting that the chosen environmental variables were effective in capturing the species' habitat preferences. Additionally, the TSS value of 0.75 further confirms the model's accuracy, indicating a strong relationship between the model and the actual distribution of *L.migratoria*. Such tests are accepted through several previous modelling works (Sarikaya et al. 2018; Hosni et al. 2020).

The contribution analysis of bioclimatic variables revealed the significant role of certain variables in shaping the species distribution. The Annual Mean Temperature (bio1) emerged as the most influential variable, accounting for 56% of the model's contribution. This finding suggests that temperature plays a crucial role in determining the suitable habitat for *L.migratoria*. The response curve further indicated that the species favours temperatures ranging between 10°C and 15°C. Other important variables include Mean Diurnal Range (bio2), Temperature Annual Range (bio7), Annual Precipitation (bio12) and Precipitation of the Driest Month (bio14), which collectively contribute to the species' distribution patterns. On the other hand, the study of closely-related species *Schistocercagregaria* has different significant influencing variables (mean temperature of coldest quarter (Bio 11); precipitation of warmest quarter (Bio 18); mean diurnal range (Bio 2); precipitation of wettest period (Bio 13) (Saha et al. 2021).

The predicted current potential distribution of *L.migratoria* aligns well with its known distribution in Europe, Africa, Asia and Australia. In Europe, the model accurately captures the species' concentration in the western regions and coastal areas surrounding the Baltic Sea, Black Sea, Mediterranean Sea, North Sea, Adriatic Sea and the Caspian Sea. This agreement with the natural distribution of the species suggests the reliability of the modelling approach (Chapuis et al. 2008).

In Africa, the model predicts very high suitability in the northern regions of the Mediterranean coasts, which lack occurrence records, but are identified as potential high-risk areas. This finding highlights the importance of considering both occurrence data and environmental variables in predicting species distributions, as the model can provide valuable insights beyond the known occurrence records. Additionally, low to medium suitability is observed in southern Africa, indicating areas of relatively lower risk. Adding the generated distribution maps to the remote sensing and satellite images of desert swarms could help to avoid economic losses caused by this species throughout the continent (Gómez et al. 2019).

The model predicts high-risk regions in Asia, particularly in Japan, southern regions of North Korea and the entirety of China. Notably, the regions surrounding the Brahmaputra River are also identified as areas of very high suitability. These findings are consistent with the known distribution and ecological behaviour of *L.migratoria*, as outbreaks often occur in the deltas of rivers flowing into seas. Medium to low suitability is observed in most parts of India, Singapore and Indonesia (Lecoq and Long 2019 ; Lecoq 2022).

In Australia, the model identifies the southeast region as a suitable habitat for *L.migratoria*, which aligns with the occurrence of the Australian plague locust, a major agricultural pest. The model also suggests low to medium habitat suitability in certain regions of New Zealand (Piou and Marescot 2023).

The study provides valuable insights into the potential impacts of climate change on the distribution of *L.migratoria*. By utilising future climate projections, researchers can assess how changes in temperature, precipitation patterns and other climatic variables may influence the range and abundance of the species. This information is crucial for developing effective strategies for locust management and implementing early warning systems to mitigate the impact on agriculture and ecosystems (Ceccato et al. 2007).

The study found that climate factors, particularly temperature and precipitation, play a crucial role in determining the distribution of *L.migratoria*. By analysing occurrence data and environmental variables, the MaxEnt models accurately predicted the potential habitat suitability of the species, demonstrating high-performance indicators. Specific bioclimatic variables, such as mean temperature and annual precipitation, were identified as significant influencers of the locusts' presence (Nasser et al. 2023). Such outcomes are very compatible with work done on the desert locust *Schistocercagregaria* (Saha et al. 2021). On the other hand, Saha et al. (2021) found that the desert locust distribution range will be shrunk due to climate change. This is different from what will happen to the migratory locust. *Locustamigratoria* has a broad distribution range, extending from Africa to Europe, Asia and Australia and has the ability to form large swarms, causing extensive damage to crops and vegetation (Hunter et al. 1999). The expansion of its distribution to different continents, especially through northern Europe, highlights its adaptability and migratory behaviour. This emphasises the importance of understanding the species' distribution dynamics and its response to climate change (Hunter et al. 1999).

Several previous studies discussed the impact of climate change on insect distribution especially for medical, veterinary and agricultural pests. In most cases, many of this species increase their distribution and invade new areas (Hosni et al. 2020, Hosni et al. 2022). The Asian tiger mosquito (*Aedesalbopictus*) is an example showing the spread of insects into new regions) . This invasive mosquito species is a known vector for several diseases, including dengue fever and chikungunya. SDM studies using MaxEnt have indicated that climate change could facilitate the further expansion of *A.albopictus* into new geographical areas. Warmer temperatures and altered precipitation patterns are expected to create more suitable conditions for the mosquito's survival and reproduction (Proestos et al. 2015). Additionally, the same thing occurs to agriculture and forest pests, such as the Pine Processionary Moth *haumetopoea pityocampa* (Roques et al. 2014). In many other cases, the climate change impact could threaten the insect species diversity and decrease their range as has occurred to the checkerspot butterfly *Euphydryasedithabayensis* in the United States (Parmesan et al. 2014) and dung beetle *Phyllognathusexcavatus* in the Mediterranean Region (Fatah et al. 2023).

The integration of GIS and MaxEnt modeling techniques provides valuable tools for predicting species distributions and assessing the potential impacts of climate change (Sabour 2023). By comparing current distribution models with future climate scenarios, researchers can evaluate how changes in temperature, precipitation patterns and other climatic variables may affect the abundance and range of *L.migratoria*. This information is crucial for identifying areas at higher risk of locust outbreaks and implementing proactive measures for effective locust management of any other agriculture pest that is studied by this methodology (Abutaleb et al. 2020).

## Conclusions

In conclusion, our study utilised Geographic Information System (GIS) and MaxEnt ecological modelling techniques to assess the ecological consequences of climate change on the distribution patterns of *Locustamigratoria*, a significant grasshopper species known for its destructive impact on crops and vegetation. Through comprehensive data analysis and predictive modelling, we identified the crucial role of climate factors, particularly temperature and precipitation, in determining the locust's distribution. Our high-performance MaxEnt models accurately predicted the potential habitat suitability of *L.migratoria*, highlighting the importance of mean temperature and annual precipitation as significant factors influencing the species' presence. Furthermore, our research generated future distribution maps that provide insights into the potential invasion of *L.migratoria* into new regions, particularly in Europe, as a direct consequence of a warming world. These findings have significant implications for agricultural communities, as they predict the risk posed by this destructive species. By shedding light on the complex relationship between locust distribution and environmental factors, our study contributes to the development of effective strategies for locust management and early warning systems. The identification of limiting factors and the mapping of ecological constraints using Diva-GIS software offer valuable tools for understanding and mitigating the impact of locust outbreaks on agriculture and ecosystems. Overall, our research provides valuable insights into the ecological consequences of climate change on *L.migratoria* and emphasises the need for proactive measures to address the challenges posed by this species. By integrating climate change considerations into locust management strategies, we can develop more effective approaches to mitigate the impact on agriculture and ensure the sustainability of ecosystems in the face of a changing climate.

## Ethics statement

The present work did not need any ethical statement as it only dealt with Data Science.

## Supplementary Material

5E5000C9-65EF-5DC2-A9A3-06A8A2309A53 10.3897/BDJ.12.e115845.suppl1 Supplementary material 1 The receiver operating characteristic (ROC) curve for Locustamigratoria Data type Graph File: oo_931750.pdf https://binary.pensoft.net/file/931750 Authors of Manuscript

## Figures and Tables

**Table 1.** Relative percentages of bioclimatic covariates used in Maxent to model the current and future habitat suitability of *L.migratoria*.

Bioclimatic variables	Description	Contribution percentages
Bio 1	Annual Mean Temperature	56%
Bio 2	Mean Diurnal Range (Mean of monthly max temp – min temp)	24.9 %
Bio 12	Annual Precipitation	7.9%
Bio 14	Precipitation of Driest Month	7.1%
Bio 7	Temperature Annual Range	4.2 %
