# Supplementary figures and images for: Locustamigratoria (L.) (Orthoptera) in a warming world: unravelling the ecological consequences of climate change using GIS
Source: Biodivers Data J. 2024 Mar 5;12:e115845. doi: 10.3897/BDJ.12.e115845 (PMC10933582; doi:10.3897/BDJ.12.e115845)

**Figure S1:** The receiver operating characteristic (ROC) curve for *Locusta migratoria*

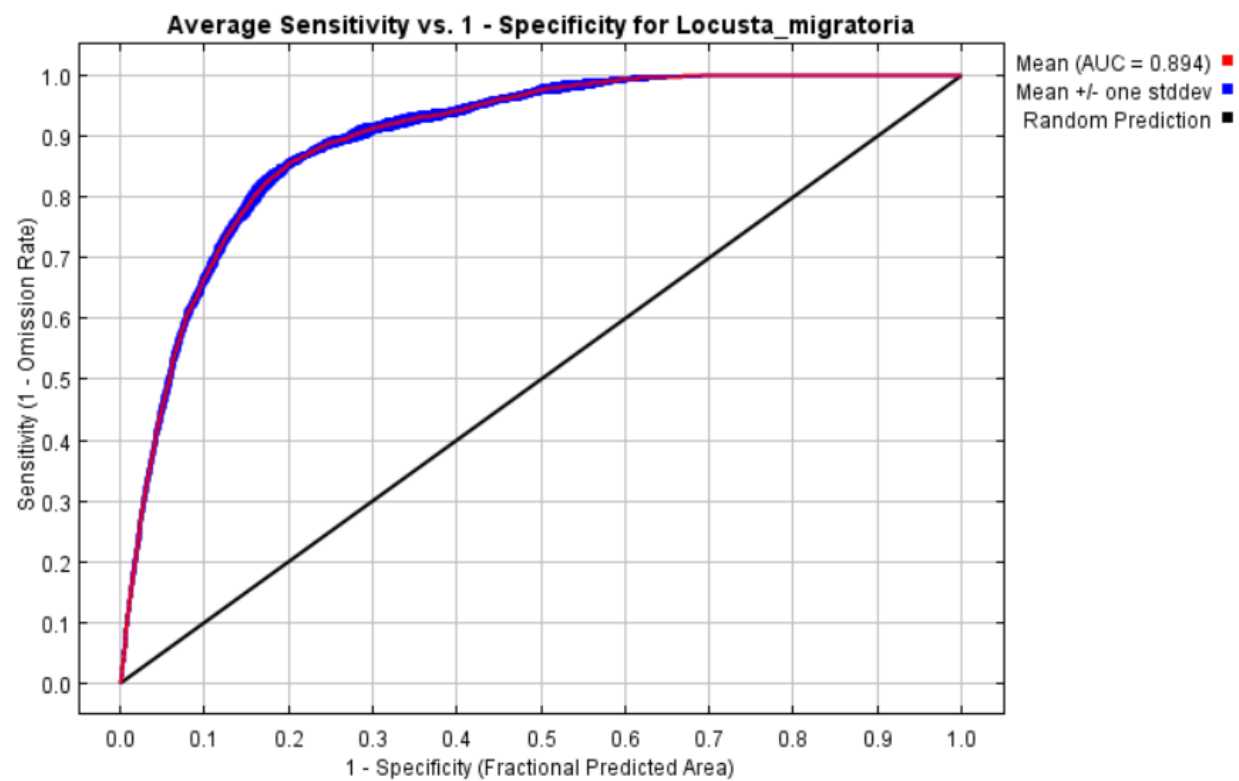

Supplement: Supplementary material 1 — The receiver operating characteristic (ROC) curve for Locustamigratoria [file bdj-12-e115845-s001.pdf]
